# 

**Published:** 1885-05

**Authors:** 


					﻿/hole Number, 3O5.
MAY, 1885.
__
M	5.
THE
CHICAGO MEDICAL- JOURNAL AND EXAMINER
EDITORS:
S. J. JONES, M.D., LL.D.
W. W. JAGGARD, A.M , M.D.
HAROLD N MOYER, M.D.
CHICAGO:
PUBLISHED MONTHLY BY THE CHICAGO MEDICAL PRESS ASSOCIATION,
240 Wabash Avenue.
CLARK & LONGLEY, PRINTERS, 163 AND 165 DEARBORN STREET, CHICAGO.
				

